# Correction to: Serial changes of the side-branch ostial area after single crossover stenting with kissing-balloon inflation

**DOI:** 10.1007/s10554-023-02936-5

**Published:** 2023-09-16

**Authors:** Tatsuhiro Fujimura, Takayuki Okamura, Ryoji Nagoshi, Yoshinobu Murasato, Masahiro Yamawaki, Yosuke Miyazaki, Hideaki Akase, Shiro Ono, Takeshi Serikawa, Yutaka Hikichi, Hiroaki Norita, Fumiaki Nakao, Tomohiro Sakamoto, Toshiro Shinke, Junya Shite

**Affiliations:** 1 Division of Cardiology, Department of Medicine and Clinical Science, Yamaguchi University Graduate School of Medicine, 1-1-1 Minamikogushi, Ube, Yamaguchi, Ube, 755-8505 Japan; 2 Department of Cardiology, Osaka Saiseikai Nakatsu Hospital, Osaka, Japan; 3 Department of Cardiology, National Hospital Organization Kyusyu Medical Center, Fukuoka, Japan; 4 Department of Cardiology, Saiseikai Yokohama City Eastern Hospital, Yokohama, Japan; 5 Department of Cardiology, Saiseikai Yamaguchi General Hospital, Yamaguchi, Japan; 6 Department of Cardiology, Fukuoka Wajiro Hospital, Fukuoka, Japan; 7 Department of Cardiology, Saga-Ken Medical Center Koseikan, Saga, Japan; 8 Department of Cardiology, Oda Hospital, Kashima, Japan; 9 Department of Cardiology, Yamaguchi Grand Medical Center, Hofu, Japan; 10 Department of Cardiology, Saiseikai Kumamoto General Hospital, Kumamoto, Japan; 11 Division of Cardiology, Department of Medicine, Showa University School of Medicine, Tokyo, Japan

**Correction to: The International Journal of Cardiovascular Imaging (2023) 39:1593–1603** **https://doi.org/10.1007/s10554-023-02853-7**

The article “Serial changes of the side-branch ostial area after single crossover stenting with kissing-balloon inflation”, was originally published Online First without Open Access. After publication in volume 39, issue 8, page the author decided to opt for Open Choice and to make the article an Open Access publication. Therefore, the copyright of the article has been changed to © The Author(s) 2022 and the article is forthwith distributed under a Creative Commons Attribution 4.0 International License, which permits use, sharing, adaptation, distribution and reproduction in any medium or format, as long as you give appropriate credit to the original author(s) and the source, provide a link to the Creative Commons licence, and indicate if changes were made. The images or other third party material in this article are included in the article’s Creative Commons licence, unless indicated otherwise in a credit line to the material. If material is not included in the article’s Creative Commons licence and your intended use is not permitted by statutory regulation or exceeds the permitted use, you will need to obtain permission directly from the copyright holder. To view a copy of this licence, visit https://creativecommons.org/licenses/by/4.0. The original article has been corrected.

